# The relationship between personality traits and Alzheimer’s disease: a narrative review

**DOI:** 10.3389/fneur.2026.1757617

**Published:** 2026-05-21

**Authors:** A. Bednorz, D. Religa

**Affiliations:** 1 John Paul II Geriatric Hospital, Katowice, Poland; 2 Institute of Psychology, Humanitas University, Sosnowiec, Poland; 3 Department of Neurobiology, Care Sciences and Society, Division of Clinical Geriatrics, Karolinska Institutet, Stockholm, Sweden; 4 Theme Inflammation and Aging, Karolinska University Hospital, Huddinge, Sweden

**Keywords:** Alzheimer’s disease, conscientiousness, dementia, neuroticism, personality traits

## Abstract

The relationship between personality traits and Alzheimer’s disease (AD) has received increasing attention in recent research. A narrative literature search was conducted in PubMed (2014–2025) using predefined keywords, with studies selected based on relevance and methodological quality. Personality traits—particularly high neuroticism and low conscientiousness—have been consistently associated with an increased risk of AD, influencing both its onset and clinical progression. Neuroticism emerges as the most robust and consistent risk factor, linked to accelerated cognitive decline, increased risk of mild cognitive impairment (MCI), and dementia through biological, psychological, and behavioral pathways. In contrast, conscientiousness appears to exert a protective effect, being associated with reduced risk of cognitive decline and greater cognitive resilience, likely mediated by health-related behaviors and neurobiological mechanisms. Extraversion and openness show more variable and context-dependent associations, with some evidence suggesting protective roles through social engagement and cognitive reserve, whereas agreeableness demonstrates weak and inconsistent relationships with AD risk. Longitudinal studies provide the strongest evidence, indicating that personality traits act as premorbid risk or protective factors rather than solely reflecting disease-related changes, while cross-sectional findings primarily capture the clinical phenotype of AD. Overall, personality traits contribute to individual differences in vulnerability to cognitive decline, highlighting their potential utility in early risk identification and prevention strategies.

## Introduction

People with dementia (PwD) may exhibit personality changes that sometimes precede other early clinical signs of the disease, such as cognitive decline. Early recognition of such changes can facilitate quicker identification of dementia (1). Evidence suggests that certain personality traits may serve as risk factors for Alzheimer’s disease (AD). The literature indicates relationships between AD neuropathology and personality (2–5). Studies have also shown that personality traits present before disease onset can influence the course of the disease or the manifestation of its symptoms (6, 7). However, the precise relationship between these personality shifts and memory impairment—a common preclinical symptom of many age-related neurodegenerative diseases—remains a topic of debate (5, 8–10).

The aim of this study was to review the literature on the relationship between personality and AD. This is a narrative review that examines various dimensions of personality and their associations with cognitive impairment, and biomarkers. Building on prior reviews (8, 11), this paper adopts a personality-centered perspective, beginning with an examination of each major personality dimension individually; however, this division is made solely for the purposes of the present article, as in reality these dimensions should also be evaluated in relation to one another, a point that has been supported by findings from previous research. The study is structured in three parts. First, we provide a focused examination of individual personality dimensions. Second, we examine the role of personality in modifiable risk factors for dementia, including health behaviors, lifestyle, and disease processes. Third, we extend this analysis to higher-order metatraits—Stability (low Neuroticism, high Agreeableness, and Conscientiousness) and Plasticity (Extraversion and Openness/Intellect)—considered as broader regulatory dimensions that may reflect shared neurobiological mechanisms relevant to AD risk and adaptation. Finally, we incorporate evidence at the facet level to provide a more fine-grained understanding of how specific components of personality dimensions contribute to cognitive aging and dementia risk. In this perspective, personality traits and higher-order metatraits can be considered in relation to approach–avoidance and stress-regulation systems. This integrative perspective links personality to HPA-axis activity, allostatic load, and fronto-limbic resilience, offering a more mechanistic account of vulnerability and progression in AD. We also refer to Gray’s Reinforcement Sensitivity Theory (RST) to broaden the theoretical context beyond the Big Five framework.

Although the review is not systematic in nature, a structured approach to literature identification and selection was applied to enhance transparency and reproducibility.

A comprehensive search of the PubMed database was performed using the following query: [“Alzheimer Disease” (MeSH) OR “Alzheimer’s disease” OR dementia] AND [“Personality”(MeSH) OR “personality traits” OR neuroticism OR extraversion OR openness OR agreeableness OR conscientiousness]. The search was restricted to articles published between 2014 and 2025 and limited to selected study types, including clinical trials, systematic reviews, narrative reviews, meta-analyses, and randomized controlled trials.

**Figure 1:**
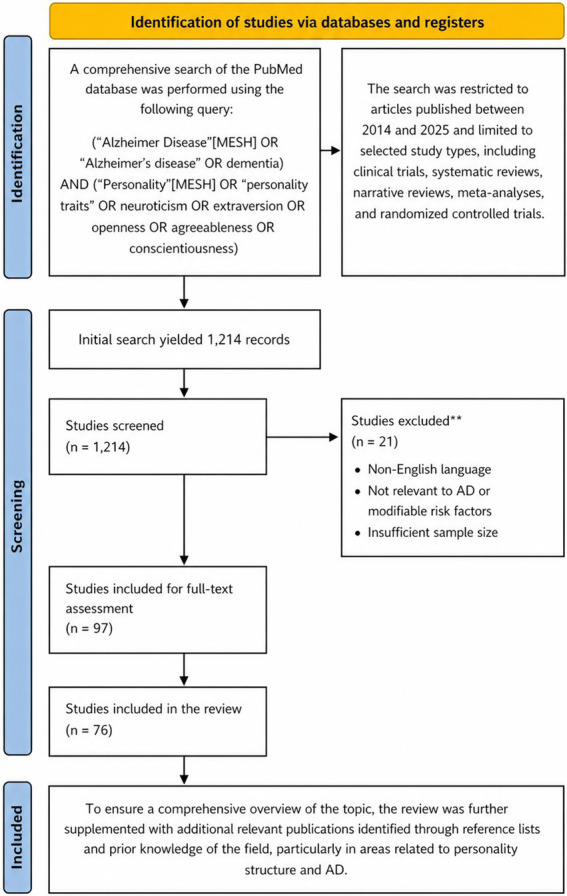
Literature search and selection strategy.

The initial search yielded 1,214 records. Titles and abstracts were screened for relevance, resulting in the inclusion of 76 studies. A total of 21 studies were excluded based on predefined criteria, including: (1) non-English language, (2) lack of direct relevance to Alzheimer’s disease or related modifiable risk factors, and (3) insufficient sample size (see Figure 1). To ensure a comprehensive overview of the topic, the review was further supplemented with additional relevant publications identified through reference lists and prior knowledge of the field, particularly in areas related to personality structure and AD.

### Alzheimer’s disease—short overview

In Europe, the incidence of clinically defined AD is approximately 3.4 per 1,000 person-years at ages 65–74, increasing three- to fourfold per decade to around 36 per 1,000 person-years after age 85. Globally, more than 57 million people were living with dementia in 2021 (60–70% attributable to AD), and this number is projected to triple by 2050; current estimates suggest that approximately 101 million individuals worldwide exhibit cognitive impairment related to AD pathology [32 million with dementia and 69 million with mild cognitive impairment (MCI)] (12). The amyloid hypothesis, one of the earliest theories related to AD, posits that the disease is initiated by the accumulation of amyloid-*β* (Aβ) in the brain, resulting from an imbalance between its production and clearance (13). Neuroimaging techniques, such as positron emission tomography (PET) and cerebrospinal fluid (CSF) analyses, reveal that Aβ accumulation may occur even a decade before the onset of clinical symptoms (14, 15). While the amyloid hypothesis has dominated AD research for over two decades, recent evidence highlights tau protein, rather than Aβ, as central to disease progression. The tau hypothesis suggests that AD advances primarily due to tau pathology, which may occur independently or alongside Aβ accumulation (16). Some researchers view AD as a tauopathy facilitated by amyloid, as tau pathology closely correlates with neuronal dysfunction and degeneration. Pathological tau can spread between cells in a prion-like manner, propagating through the brain in a specific spatiotemporal pattern that aligns with clinical symptoms and disease progression (17). Systemic infections and associated inflammatory responses can accelerate amyloid pathology, while the diseased brain becomes increasingly vulnerable to further inflammation, creating a self-perpetuating cycle of neurodegeneration (18).

### Historical overview

Personality traits are increasingly recognized as predictors of late-life cognitive outcomes, influencing cognitive trajectories beyond biological and lifestyle factors. As early as Botwinick (19), personality was proposed to contribute to cognitive reserve, with disturbances in personality functioning potentially precipitating cognitive deficits—an idea partially reflected in the DSM-5 (20, 21), which conceptualizes personality pathology in terms of impairments in self-regulation and goal-directed behavior, closely related to cognitive control and flexibility. Developmental research supports this link: temperament, as the biological basis of personality, relates to early information processing and self-regulation. Rothbart’s model identifies effortful control—attentional focusing, inhibitory control, and perceptual sensitivity—as central to executive functions (22), and longitudinal findings show that early self-regulation predicts later cognitive control (23). Overall, personality traits shape engagement in cognitively stimulating activities, health behaviors, and social interactions that build cognitive reserve. Conscientiousness predicts academic and cognitive performance independently of intelligence and may enhance long-term cognitive resilience despite neurodegeneration (24–26).

Research on the relationship between cognitive impairment and personality traits began in the 1980s and 1990s. Early studies on personality changes and dementia were conducted retrospectively, relying on reports from family. These studies indicated progressive personality changes as the disease advanced, including increased neurotic traits, decreased extraversion and conscientiousness, and reduced openness and agreeableness (27).

Among various personality models, the Five-Factor Model (FFM) was identified as particularly useful for diagnosing personality traits (6). The primary research tool, still widely used today, is the Revised NEO Personality Inventory (NEO-PI-R), developed by Costa and McCrae (28). Table 1 presents the fundamental personality dimensions of the Big Five model as conceptualized by Costa and McCrae, together with the higher-order metatraits of Stability and Plasticity (29). The table summarizes their core descriptive features as well as their functional significance in terms of goal regulation, exploration, and behavioral control. These constructs provide the theoretical framework for the subsequent sections of this paper, in which their neurobiological will be discussed in detail (see Tables 1, 2). In U.S.-based studies (30, 31), neuroticism—also referred to as “proneness to psychological distress” (32)—was typically measured using the NEO-PI. An exception was the Chicago Health and Aging Project (CHAP) (33), which assessed “cynical distrust” using a subset of the Cook-Medley Hostility Scale (34). This same scale was also employed in the Cardiovascular Risk Factors, Aging, and Dementia (CAIDE), study conducted in Scandinavia (35). However, the CAIDE study focused on dementia diagnoses over an average follow-up of 8.4 years, while the CHAP study primarily evaluated global cognitive function at baseline and cognitive decline over 4.4 years. Similarly, the Swedish Twin Registry Study assessed cognitive decline over a 25-year period, using the Eysenck Personality Inventory (EPI-Q) (36) to measure neuroticism and extraversion as key predictors (37).

**Table 1:** Characteristics of personality dimensions (28, 29).

Level	Dimension/Trait	Core features/Facets	Function	Negative pole
Metatraits	Stability (CON+AGR + NEU ↓)	Emotional and motivational regulation	Protection of goals, interpretations, and strategies from disruption by impulses	Unstable
	Plasticity (EXT + OPE)	Exploratory and adaptive tendencies	Exploration: creation of new goals, interpretations, and strategies	Rigid
Big Five	NEU	Anxiety Angry Hostility Depression Self-Consciousness Impulsiveness Vulnerability	Defensive responses to uncertainty, threat, and punishment	Unflappable
	EXT	Warmth Gregariousness Assertiveness Activity Excitement-Seeking Positive Emotions	Behavioral exploration and engagement with specific rewards	Reserved
	OPE	Fantasy Aesthetics Feelings Actions Ideas Values	Cognitive exploration and engagement with information	Unimaginative
	AGR	Trust Straightforwardness Altruism Compliance Modesty Tender-Mindedness	Altruism and cooperation; coordination of goals and strategies with others	Selfish
	CON	Competence Order Dutifulness Achievement Striving Self-Discipline Deliberation	Protection of non-immediate or abstract goals from disruption	Unreliable

NEO trait abbreviations: NEU, Neuroticism; EXT, Extraversion; OPE, Openness to experience; AGR, Agreeableness; CON, Conscientiousness.

**Table 2:** Personality dimensions of the big five and their neurobiological correlates.

Dimension	Key brain structures and additional neurobiological findings*
EXT	Dopamine (“wanting”), endogenous opioids (“liking”) (144) ↑ temporal regions, dlPFC, ACC, insula (238) ↓ parahippocampal, occipital, parietal regions (238) ↑ SMA, lingual gyrus, inferior parietal lobule (87) ↓ middle temporal gyrus, superior frontal gyrus (87) associated with volume of ventromedial prefrontal cortex (29) fMRI: increased activity to rewards (29) EEG: reward-dependent arousal modulation (29, 87) pharmacology: D2 receptor sensitivity (agentic Extraversion modulates response to D2 antagonism) (86, 145)
NEU	↑ occipital, fusiform (238) ↓ orbitofrontal, dlPFC, parahippocampal, temporal regions (238) ↑ activity in threat- and punishment-related regions (amygdala, insula, anterior cingulate cortex) and altered medial prefrontal cortex emotion regulation (29) ↓ medial prefrontal cortex volume (129) HPA axis involvement (65) ↑ affective reactivity, serotonergic polymorphisms (e.g., 5-HTTLPR) and fronto-limbic emotional regulation circuits (86, 140) genetic vulnerability (50)
AGR	↑ dmPFC, ACC, hippocampus, caudate (238) ↓ frontopolar, temporal, parietal, orbitofrontal regions (238) ↑ volume in regions involved in understanding others’ intentions and mental states (mentalizing/social cognition networks) (86) ↑ prefrontal cortex activity during emotion regulation (fMRI); associated with suppression of aggressive and socially disruptive emotions (29)
CON	↑ dmPFC, ACC, hippocampus, caudate (238) ↓ frontopolar, temporal, parietal, orbitofrontal regions (238) ↑ white matter integrity (fractional anisotropy) in the left uncinate fasciculus (141) ↑ lateral prefrontal cortex volume (planning and voluntary control of behavior) (86) ACC & insula (compassion-related subdimension) (110)
OPE	Dopamine (PFC), COMT (144) ↑ frontopolar cortex (BA10), thalamus (238) ↓ orbitofrontal, fusiform, fronto-insular, parietal regions (238) associated with IQ (29) predicts global efficiency of default network (143)

Arrows (↑/↓) indicate the direction of associations with regional gray matter (GM) volume (MRI-based morphometry), with ↑ denoting greater volume and ↓ denoting lower volume in relation to the respective personality trait. dlPFC, dorsolateral prefrontal cortex; dmPFC, dorsomedial prefrontal cortex; ACC, anterior cingulate cortex; BA10, Brodmann area 10; HPA, hypothalamic–pituitary–adrenal axis; EEG, electroencephalography; fMRI, functional magnetic resonance imaging; D2, dopamine type 2 receptor; COMT, catechol-O-methyltransferase; PFC, prefrontal cortex; SMA, supplementary motor area. NEO trait abbreviations: NEU, Neuroticism; EXT, Extraversion; OPE, Openness to experience; AGR, Agreeableness; CON, Conscientiousness. *These data are derived from a study conducted in healthy older adults from the Baltimore Longitudinal Study of Aging (BLSA) and were included given the explicit reference to this study in the present article (238).

Table 2 summarizes the Big Five personality dimensions, key neurobiological systems, and neuroimaging correlates, providing a framework for understanding their underlying mechanisms.

### Neuroticism

Among the Big Five personality traits, neuroticism consistently emerges as a significant vulnerability factor in cognitive aging and AD. High neuroticism—characterized by emotional instability, heightened reactivity to stress, and chronic negative affect—has been linked to accelerated cognitive decline, increased risk of MCI, and higher incidence of dementia (31, 38, 39). This association is thought to operate through multiple pathways, including biological stress mechanisms, behavioral disengagement, and neurobiological vulnerability (11).

Based on a systematic review, higher neuroticism is the most consistently identified risk factor in longitudinal studies, predicting increased likelihood of MCI, dementia, and faster cognitive decline, particularly in interaction with genetic risk (e.g., apolipoprotein E -APOE ε4), although some studies report null findings (40). In the Baltimore Longitudinal Study of Aging (*n* = 1,671, follow-up up to 22 years), higher neuroticism increased AD risk by 30% (HR = 1.37), while high conscientiousness, openness, agreeableness, and extraversion reduced it (39). Similar findings emerged from ELSA (*n* = 6,887) and HILDA (*n* = 2,778), showing neuroticism predicts dementia onset (38). In a longitudinal Swedish Twin Study of 4,039 individuals followed over 25 years, higher neuroticism was associated with increased risk of cognitive impairment, while moderate extraversion appeared protective, highlighting complex interactions between these traits (37). In a longitudinal study with a 38-year follow-up, higher midlife neuroticism was associated with increased risk of AD, partly mediated by long-term distress, whereas extraversion was linked to lower distress but not directly to AD risk; the highest risk was observed in individuals with high neuroticism and low extraversion (41). Complementary findings from a 6-year population-based cohort study (*n* = 506; Kungsholmen Project) showed that low neuroticism combined with high extraversion was associated with the lowest dementia risk, particularly among socially inactive individuals, underscoring the role of personality–lifestyle interactions in cognitive aging (42). In a cohort of 174,164, higher neuroticism raised risk of all-cause dementia by 11% and vascular dementia by 15%, partly mediated by mental and vascular conditions, and linked to cerebrovascular pathology, reduced gray matter, and poorer cognition (43). Finally, longitudinal evidence suggests that personality itself may change in the context of cognitive decline. A large study from the Health and Retirement Study (*n* = 22,611) showed that increases in neuroticism and declines in other traits occur more rapidly during cognitive impairment, whereas preclinical personality changes are relatively small and inconsistent (10). This suggests that neuroticism may function both as a vulnerability factor and, in some cases, as a prodromal marker of emerging pathology.

In MCI, longitudinal evidence indicates that individuals with MCI show increases in neuroticism and decreases in conscientiousness and extraversion, while no significant changes are observed in controls (44). Additionally, another longitudinal study (*n* = 222) found that higher baseline neuroticism more than doubled the risk of MCI, with affected individuals consistently showing higher neuroticism and lower openness than healthy controls (45). Other longitudinal findings are mixed: one cohort study (*n* = 2046; up to 36 years of follow-up) found no significant personality changes prior to MCI or dementia onset, arguing against their role as early markers (46). In contrast, another longitudinal study (*n* = 210 dementia; 135 MCI; 1,740 controls) reported increases in neuroticism before diagnosis, suggesting it may serve as a potential early indicator of cognitive decline (47). Furthermore, in a longitudinal study (*n* = 1954), higher neuroticism was associated with an increased risk of transition from no cognitive impairment (NCI) to MCI (HR = 1.12), indicating its role as a risk factor in early cognitive decline (48).

Genomic studies show neuroticism is a stable behavioral marker of general genetic vulnerability across psychopathologies (49). At the genetic level, neuroticism represents a highly heritable and biologically complex trait. Large-scale genomic studies indicate that it is associated with hundreds of loci involved in neurogenesis, axonal function, and dopaminergic and serotonergic signaling, suggesting multiple, partly distinct biological pathways (50). This heterogeneity is further supported by findings that specific facets of neuroticism—such as anxiety, depression, impulsivity, and vulnerability—have partially distinct genetic architectures linked to pathways including axon guidance (51). Earlier Genome-Wide Association Studies (GWAS; Genome-Wide Association Studies) identified loci such as SCAMP2 (Secretory Carrier Membrane Protein 2), KATNAL2 (Katanin Catalytic Subunit A1 Like 2), XKR6 (X Kell Blood Group Complex Subunit Related 6), L3MBTL2 [Lethal(3) Malignant Brain Tumor-Like Protein 2], and CHADL (Chondroadherin Like), which are implicated in synaptic signaling and neuronal development (52). Candidate gene studies suggest more nuanced effects: for example, BDNF (Brain-Derived Neurotrophic Factor) Val66Met polymorphism has been linked more strongly to introversion, whereas variation in 5-HTTLPR (serotonin-transporter-linked polymorphic region in the SLC6A4 gene) may modulate levels of neuroticism (53). In the DIAN study (Dominantly Inherited Alzheimer’s Network), carriers of autosomal dominant Alzheimer’s disease mutations in PSEN1 (Presenilin 1), PSEN2 (Presenilin 2), and APP (Amyloid Precursor Protein) tended toward higher neuroticism and lower conscientiousness, with CSF tau correlating with these traits (54). In a longitudinal study of 602 older adults followed for 6.5 years, personality traits were found to moderate the relationship between APOE ε4 and cognitive outcomes, with genetic risk being evident primarily among individuals high in neuroticism and extraversion, but not among those with lower levels of these traits (55). Importantly, environmental influences interact with genetic vulnerability, as large meta-analytic evidence shows that childhood trauma—especially emotional neglect and abuse—is associated with elevated neuroticism in adulthood (56).

In the context of AD biomarkers, neuroticism appears to play a complex and interaction-dependent role, influencing cognitive functioning in relation to underlying pathological burden rather than exerting a uniform effect. Studies have shown that higher neuroticism and lower extraversion, openness, and conscientiousness are associated with reduced Aβ42, indicating relationships between personality changes and early amyloid pathology (2). Higher neuroticism was associated with worse cognitive functioning only at relatively low levels of CSF tau and tau/Aβ42 ratios, suggesting that its detrimental effect is most evident in the absence of pronounced AD pathology. This pattern further indicates a possible “cutoff point” of cerebral pathology, after which the interaction between neuroticism and biomarkers on cognitive performance may reverse (3).

Neuroimaging studies using PET show that higher neuroticism is associated with increased tau deposition in the entorhinal cortex, inferior temporal regions, and amygdala, as well as with smaller brain volumes and steeper age-related decline (57, 58). Additionally, a longitudinal study (*n* = 436 controls; *n* = 74 early AD) found that higher neuroticism and lower conscientiousness discriminate early AD, with their effects on dementia partly mediated by hippocampal volume (59). Although the amygdala is affected early in AD and linked to affective symptoms, its specific structural association with neuroticism remains mixed (60–62). Importantly, the amygdala consists of functionally distinct nuclei, so future research should focus on specific subregions (63). Greater white matter hyperintensities (WMH) burden was associated with higher neuroticism and lower conscientiousness, while no associations were found for MTL atrophy or lacunar strokes. These findings suggest that personality changes in MCI are more closely linked to MRI-derived white matter pathology than to medial temporal lobe degeneration (64).

The results indicate that in older adults, higher neuroticism is associated with hypothalamic–pituitary–adrenal (HPA) axis dysregulation, which may contribute to adverse health outcomes (65). Individuals high in neuroticism tend to exhibit heightened stress reactivity, leading to chronic activation of the HPA axis and autonomic nervous system, disruption of allostasis, and ultimately cognitive impairment (40). Furthermore, chronic glucocorticoid exposure in adulthood has been linked to depressive disorders (66), which are modifiable risk factors for dementia (67). In the ADNI study (The Alzheimer’s Disease Neuroimaging Initiative) (*n* = 304; MCI cohort), higher cortisol levels predicted faster hippocampal atrophy, while reduced hippocampal volume increased the risk of progression to AD, highlighting the role of HPA-axis dysregulation in neurodegeneration (68). Dysregulation of the HPA axis has also been linked to heightened stress sensitivity and may contribute to aggressive behavior (69). In addition, neuroticism has been linked to systemic inflammation and altered glucocorticoid signaling (66), suggesting a role of chronic stress and immune dysregulation (70). Consistent with this, both neuroticism and negative affect have been associated with increased dementia risk through inflammatory pathways, with evidence pointing to a bidirectional relationship between inflammation and psychological processes (71).

At the cognitive and psychological level, neuroticism is consistently associated with poorer cognitive functioning. Higher neuroticism is linked to greater subjective cognitive complaints (72) and poorer memory performance, as demonstrated in large-scale longitudinal meta-analyses (73). Moreover, higher neuroticism—particularly when combined with lower conscientiousness—is associated with accelerated memory decline over time (73). Converging evidence also indicates that neuroticism and perceived stress are associated with cognitive decline, especially in memory domains, highlighting their role as potentially modifiable risk factors (74).

Beyond cognition, neuroticism is strongly linked to broader psychological vulnerability. It is a robust risk factor for geriatric depression and maladaptive coping (75), and is associated with altered affect regulation, including patterns of affect balance that depend on current emotional states (76). Individuals high in neuroticism also tend to report an older subjective age, which has been linked to poorer health outcomes (77). These factors may further amplify dementia risk through behavioral and physiological pathways.

Collectively, this evidence positions neuroticism as a multifaceted vulnerability factor operating across genetic, neurobiological, psychological, and behavioral levels. Rather than directly reflecting neuropathology, neuroticism appears to influence cognitive aging primarily through systemic, emotional, and lifestyle-related mechanisms, making it a key target for early identification and intervention. Neuroticism is a heterogeneous construct encompassing multiple facets, sometimes with divergent effects (78, 79). Consistently, neuroticism has been identified as a general risk factor for a wide range of mental health problems, including depression, anxiety, sleep disturbances, and psychotic disorders (79–82). Personality traits are strongly associated with mental disorders, with neuroticism emerging as the most robust and consistent correlate, alongside lower conscientiousness and extraversion (83).

### Extraversion

Extraversion plays a more complex and nuanced role in cognitive aging and the risk of AD. Unlike neuroticism, whose negative impact on cognitive functioning is well documented, the effects of extraversion appear to depend on interactions with other personality traits, contextual factors, and life stage.

Based on a systematic review, extraversion shows generally weak and inconsistent associations, with some evidence suggesting a protective role (e.g., lower dementia risk and greater likelihood of cognitive recovery), while other findings indicate increased risk in genetically vulnerable individuals (40). A meta-analysis of the Baltimore Longitudinal Study of Aging data reported that higher extraversion was associated with a 5% reduction in AD risk, though this effect was smaller than for conscientiousness or openness and persisted after controlling for health and lifestyle factors (39). In a prospective study, Terracciano et al. (46) found that lower extraversion in early adulthood predicted higher dementia incidence later in life, with no significant changes in this trait prior to diagnosis—supporting the conceptualization of personality as a stable risk factor rather than an early symptom of disease. In the Swedish Twin Registry (*n* = 4,039), moderate extraversion was associated with a lower risk of cognitive impairment over 25 years, whereas the combination of high neuroticism and low extraversion significantly increased the likelihood of developing dementia (37). The results indicate that extraversion alone was not directly associated with dementia risk; however, in combination with low neuroticism—particularly among socially isolated individuals—higher extraversion was linked to reduced risk, highlighting its context-dependent and interactive role (42). In a study (*n* = 381), extraversion—alongside openness—predicted cognitive abilities in older adults, particularly those with average cognitive performance, although the pattern of associations differed from that observed in younger individuals (84). D’Iorio et al. (85) confirmed that individuals with AD exhibit lower extraversion compared to healthy controls, reflecting both the impact of neurodegenerative processes and reduced social engagement in the preclinical stages.

At the neurobiological level, extraversion is associated with activity in reward-related and dopaminergic cortical networks, including the anterior cingulate and medial prefrontal cortex (29, 86). Furthermore, a systematic review and meta-analysis of resting state functional magnetic resonance imaging studies (11 studies, *n* ≈ 1,200) found that extraversion is positively associated with activity in the right supplementary motor area (SMA), lingual gyrus, left inferior parietal lobule, and BA48, and negatively associated with the middle temporal gyrus and right superior frontal gyrus, pointing to the involvement of fronto-parietal networks and reward-related systems (87). Individuals with higher extraversion also show greater white matter integrity in fronto-limbic tracts, which may contribute to cognitive resilience. However, during AD progression, the weakening of these networks partially accounts for observed declines in extraversion reported by caregivers (11).

Extraversion may exert protective effects via psychological and social mechanisms. Extraverted individuals maintain broader social support networks, which are associated with lower cortisol levels and reduced depression risk—factors known to negatively affect the hippocampus and episodic memory (8). A study of 120 healthy men found that high basal cortisol levels are linked to stress resilience and higher extraversion, whereas strong stress-induced cortisol responses predispose stress-sensitive individuals to more rapid emotional recovery (88). Experience sampling studies and a meta-analysis show that the link between extraversion and positive affect is partly mediated by dynamic behavioral states, suggesting a regulatory mechanism (89). A meta-analysis of over 117,000 individuals showed that high neuroticism and low extraversion and conscientiousness predict depressive symptoms over time, while depression itself is associated with subsequent personality changes (90). These findings imply that the interplay between emotional stability and social engagement may be crucial for maintaining cognitive functions in later life.

In summary, current evidence suggests that extraversion does not act as a straightforward protective factor against AD, but moderate levels may help preserve cognitive reserve and delay symptom onset. Interactions between extraversion and other personality dimensions—particularly neuroticism and openness—appear crucial for understanding heterogeneous trajectories of cognitive aging.

### Conscientiousness

Conscientiousness is consistently identified as one of the most robust personality predictors of healthy cognitive aging and reduced risk of AD. Based on a systematic review, higher conscientiousness emerges as a robust protective factor, associated with reduced risk of cognitive decline, MCI, and dementia, and may also buffer genetic risk, although not all studies confirm this relationship (40).

Population-based studies consistently highlight the protective role of conscientiousness. In the Baltimore Longitudinal Study of Aging (*n* = 1,671), lower conscientiousness was associated with a 74% increased risk of AD (HR = 1.74), whereas higher levels reduced risk by 23% (39). Similar findings from ELSA (*n* = 6,887) and HILDA (*n* = 2,778) showed that lower conscientiousness predicts dementia risk independently of health behaviors (38). In a longitudinal study (*n* = 436 controls; *n* = 74 early AD), higher conscientiousness emerged as an independent behavioral marker of lower AD risk and a predictor of conversion, comparable to biomarkers (59). These findings are supported by a large population-based cohort (*n* > 10,000; up to 8 years follow-up), where lower conscientiousness was associated with increased risk of dementia and progression to dementia, independent of clinical and demographic factors (46). Supporting this, a longitudinal study combining annual cognitive assessments with post-mortem neuropathological examination found that individuals with higher conscientiousness experienced slower terminal cognitive decline, even after accounting for neuropathological burden (91). Longitudinal studies indicate that conscientiousness remains relatively stable across the lifespan, allowing it to function as a premorbid protective factor rather than a consequence of neurodegenerative processes (11, 46). Meta-analytic evidence further indicates that individuals with AD display lower conscientiousness than healthy controls, suggesting a premorbid vulnerability profile (85).

Higher risk of incident AD was observed in individuals scoring in the lowest quartile of conscientiousness (HR = 3.40, 95% CI 1.39–8.28), independently of APOE ε4 status (39). In the DIAN study, involving individuals with autosomal dominant AD mutations (PSEN1, PSEN2, APP), lower conscientiousness was associated with higher CSF tau levels among mutation carriers, alongside a trend toward overall reduced conscientiousness in this group (54). Importantly, higher conscientiousness was linked to slower cognitive decline, suggesting a potential protective role even in the presence of early AD-related biomarker changes. These findings indicate that conscientiousness may buffer against pathological processes indexed by CSF biomarkers in genetically at-risk individuals. A large GWAS meta-analysis (>20,000 participants) identified significant associations for conscientiousness in the brain-expressed KATNAL2 gene (Katanin Catalytic Subunit A1 Like 2), a gene involved in microtubule regulation and neuronal development, pointing to specific—though not yet fully replicated—genetic substrates of this trait (92).

In a longitudinal study (*n* = 1954), higher conscientiousness predicted a reduced risk of transition from NCI to MCI (HR = 0.78), suggesting a protective effect on cognitive health (48). Duron et al. (64) found that lower conscientiousness in patients with MCI was strongly associated with greater severity of WML, but not with medial temporal lobe atrophy or lacunar strokes. Donati et al. (44) found that lower conscientiousness was associated with MCI group membership and that individuals with MCI showed a significant decline in conscientiousness over time compared to earlier assessments, whereas no such change was observed in controls. Overall, reduced conscientiousness appears to characterize both premorbid differences and subsequent change in MCI.

In the context of AD biomarkers, at low levels of premorbid conscientiousness, higher Aβ42 and lower ptau-181/Aβ42 ratios predicted worse cognitive functioning, suggesting that higher conscientiousness may exert a protective effect against biomarker-related cognitive decline (3). Higher conscientiousness is associated with larger brain volumes and reduced age-related decline, particularly in prefrontal and medial temporal regions (57). Data from the Lothian Birth Cohort 1936 (mean age ≈73; *n* = 529–565) showed that lower conscientiousness was associated with poorer brain integrity, including greater brain tissue loss, lower white matter fractional anisotropy (FA; a diffusion tensor imaging–derived measure of white matter integrity), and higher WMH burden. These associations were partly mediated by health-related behaviors such as smoking, physical activity, diet, alcohol use, and body mass index (93). Biological mechanisms may partially explain these associations. High conscientiousness correlates with lower levels of inflammatory markers, including C-reactive protein (CRP) and interleukin-6 (IL-6), which may protect against vascular and neurodegenerative damage (94).

These results suggest that high conscientiousness, particularly in the context of self-discipline, may reduce the risk of dementia. Although personality traits do not directly moderate the link between neuropathology and dementia, high conscientiousness may protect against its clinical manifestation, supporting the cognitive resilience hypothesis (71). This protective effect may arise from conscientiousness-related behaviors that safeguard against diseases increasing AD risk, such as cardiovascular disease, diabetes, and obesity. Conscientiousness may also promote health-enhancing activities, such as physical and mental engagement, which further reduce AD risk. Therefore, conscientiousness serves as a valuable proxy for beneficial behaviors and health-related lifestyles, making it a simple yet effective marker for predicting AD risk (8, 11, 95). Behavioral genetic research suggests that conscientiousness plays a key role in healthy aging through genetic and environmental mechanisms, with higher levels buffering against adverse environmental influences such as problem alcohol use (96). In adults with type 2 diabetes, higher conscientiousness was linked to greater improvements in medication adherence and emotional burden, while self-efficacy predicted better diet and physical activity, with combined high levels producing the strongest effects (97). Evidence linking personality to longevity consistently highlights conscientiousness as a key protective factor, with higher levels associated with a longer lifespan (98).

In summary, conscientiousness represents a robust, multidimensional protective factor in cognitive aging and AD risk. Its effects are mediated through behavioral, social, and neurobiological pathways, highlighting its potential as a target for interventions aimed at promoting cognitive resilience and healthy brain aging.

### Openness to experience

Openness to experience is consistently identified as one of the most robust personality correlates with preserved cognitive functioning in older adulthood. Based on a systematic review, although several studies report no association, the overall pattern suggests that higher openness is protective against cognitive decline, MCI, and dementia, potentially reflecting its role in cognitive reserve, though effects may diminish when controlling for other traits (40). Epidemiological evidence supports the protective role of openness against AD and cognitive impairment. In the Baltimore Longitudinal Study of Aging, higher openness was associated with a 14% reduction in AD risk, independent of health and lifestyle factors, while high conscientiousness and extraversion also conferred additional, though smaller, protective effects (39). Large population studies in England (ELSA) and Australia (HILDA) corroborated these findings, highlighting openness as a consistent predictor of lower dementia risk (38). A coordinated analysis across four longitudinal cohorts also found that high openness was associated with total cognitive functions, while openness was associated with decline after dementia diagnosis (99). Meta-analyses similarly indicate that individuals with AD typically exhibit lower openness compared to healthy controls (85). In a longitudinal study of 767 older adults (≥72 years) followed for over 6 years, lower openness was independently associated with increased risk of AD, supporting its role as a potential protective factor in cognitive aging (100). However, in a longitudinal cohort of 2,046 participants (mean age ≈62.6 years), openness was not significantly associated with risk of MCI or dementia, and no differences in longitudinal change were observed between cognitively impaired and nonimpaired individuals (46). Furthermore, associations between openness and cognition or dementia are inconsistent, possibly due to cultural differences and the impact of cognitive decline on self-reported openness (71).

Openness also appears to be a relevant factor in MCI. Lower premorbid openness was associated with greater likelihood of MCI group membership (44). In a longitudinal study, individuals with MCI showed consistently lower openness across follow-up assessments compared to healthy controls (45). In a longitudinal study (*n* = 488, 18-month follow-up), higher openness to experience was associated with cognitive preservation in healthy older adults, whereas lower openness increased the risk of decline and MCI (101).

Similar to extraversion, openness shows no consistent association with AD biomarkers, particularly tau deposition. PET-based analyses found no significant relationship between openness and regional tau accumulation (58), and no substantial contribution of openness to disease conversion or progression in biomarker-informed models (54, 59). Nonetheless, openness may also weakly moderate the relationship between AD biomarkers and cognitive functioning (3). Additionally, cross-sectional findings indicate that lower openness is associated with decreased CSF Aβ42 levels, suggesting a potential link with early amyloid pathology (2). Overall, these results remain limited and inconsistent, indicating that openness may reflect personality changes accompanying early disease processes rather than serving as a direct correlation of neuropathology.

Studies examining specific cognitive domains demonstrate that openness predicts superior outcomes in memory, information processing speed, visuospatial abilities, and executive functions. Other investigations have confirmed significant positive associations between openness and verbal memory, reasoning, reading competence with several models indicating partial mediation by everyday cognitive engagement (102–105).

Through engagement in novel and socially stimulating activities, openness may enhance neural connectivity, strengthen cognitive reserve, and delay the clinical manifestation of neuropathology (11). Overall, openness to experience is consistently associated with resilience against age-related cognitive decline and neurodegeneration. Higher openness is associated with better cognitive status and greater cognitive reserve, likely because individuals high in openness engage more frequently in intellectually and socially stimulating activities, thereby promoting cognitive resilience.

### Agreeableness

Regarding the risk of cognitive impairment, agreeableness appears to play a more subtle and less consistent role than other personality traits. While higher agreeableness has been associated with reduced risk of AD and better preservation of social and emotional functioning, evidence from meta-analyses is mixed. A systematic review/meta-analysis reported no association between agreeableness and dementia, and a later meta-analysis identified only a small effect that disappeared after full adjustment (46, 106). Similarly, a large cohort/meta-analysis found that lower agreeableness was linked to increased dementia risk, although findings were notably less consistent than for neuroticism and conscientiousness (46, 107). In a long-term prospective study (*n* = 1,671; follow-up up to 22 years), alongside a meta-analysis of five studies (*n* = 5,054), agreeableness showed only a weak association with incident AD compared to stronger effects for neuroticism and conscientiousness (39). Based on a systematic review, agreeableness appears to play a relatively minor and less consistent role, with most studies showing no effect, though some evidence indicates that higher agreeableness may modestly reduce the risk of dementia and cognitive impairment (40). Taken together, these results suggest that although agreeableness is routinely included in models of dementia risk, its independent contribution is modest and not robust.

Importantly, agreeableness may be more relevant to the clinical expression of dementia than to its onset. Lower premorbid agreeableness has been consistently linked to greater agitation and irritability in individuals with AD (108), indicating an association with behavioral and emotional symptom profiles. Supporting this interpretation, research on related socio-cognitive constructs shows that AD is associated with deficits in cognitive theory of mind and cognitive empathy, while affective empathy remains relatively preserved (109). Neuroimaging findings further suggest that the compassion component of agreeableness is associated with structural variation in the anterior cingulate cortex and insula, highlighting distinct neural substrates underlying its subdimensions (110). Agreeableness may exert its influence indirectly through social and behavioral pathways. Individuals higher in agreeableness tend to exhibit greater prosocial behavior and emotional regulation, which may support cognitive reserve via enhanced social engagement and adaptive coping strategies. Finally, longitudinal evidence indicates that agreeableness tends to increase with age (111), which may reflect an adaptive developmental process potentially buffering against age-related stress. Overall, agreeableness appears to play a secondary, largely indirect role in cognitive aging—more closely linked to social functioning and behavioral manifestations of dementia than to core neuropathological processes.

**Figure 2:**
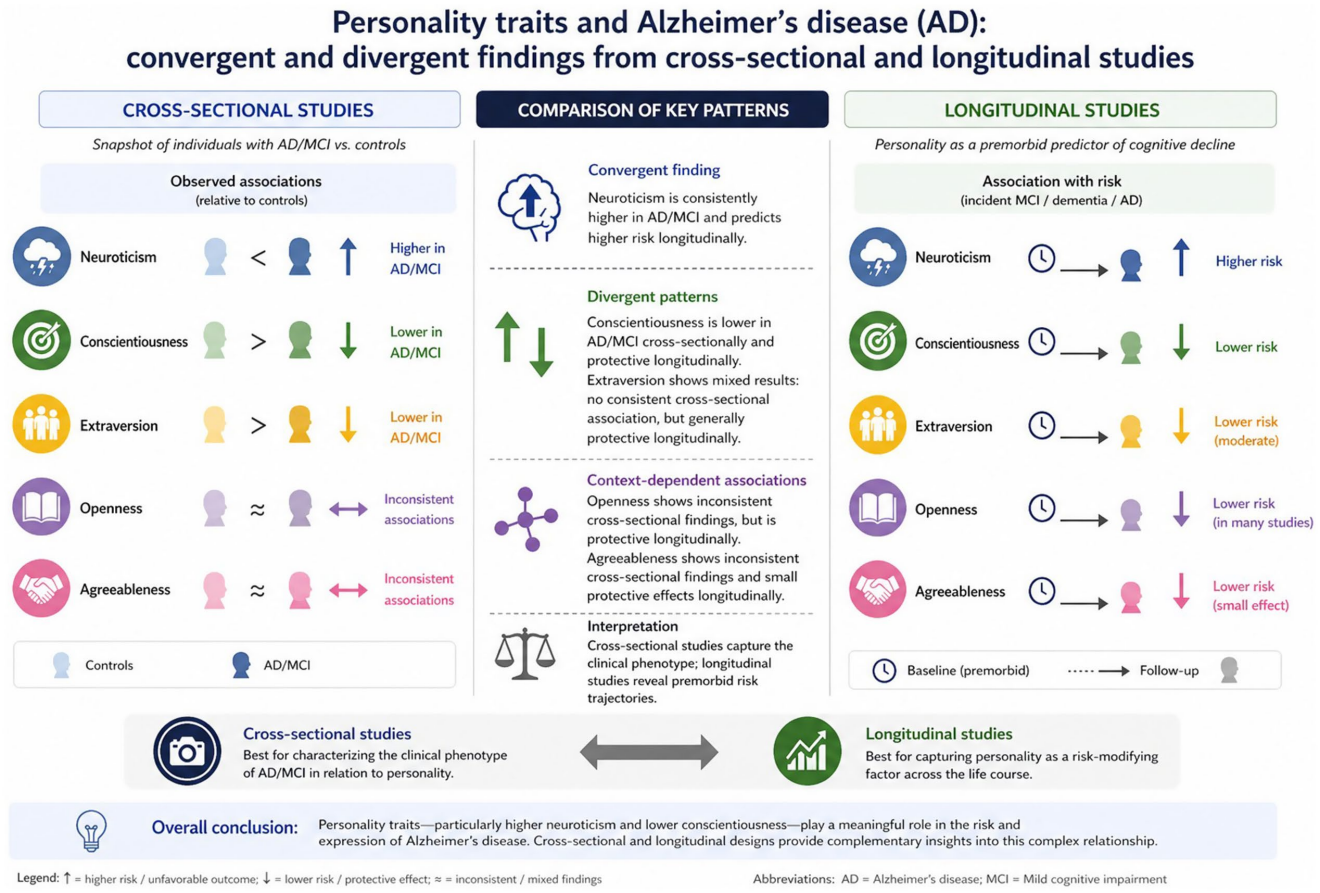
Personality and Alzheimer’s disease: insights from cross-sectional and longitudinal studies.

A summary of selected studies on the relationship between personality traits and AD is presented in Tables 3, 4. A comparison of findings from cross-sectional and longitudinal studies reveals both convergent and divergent patterns in the association between personality traits and risk of AD. Cross-sectional studies generally show concurrent associations between personality traits and cognitive status, with higher Neuroticism and lower Openness and Conscientiousness observed in individuals with MCI or AD compared to controls, while effects for Extraversion and Agreeableness are less consistent. In contrast, longitudinal studies consistently demonstrate that higher baseline Neuroticism predicts increased risk of incident MCI, dementia, or AD over time, whereas higher Conscientiousness—and in some studies also Openness and Extraversion—shows a protective effect or reduced risk of cognitive decline across follow-up periods. While cross-sectional designs primarily capture concurrent associations that may reflect both premorbid vulnerability and disease-related changes, longitudinal evidence supports a directional interpretation in which personality traits contribute to long-term risk trajectories. Taken together, these findings suggest that cross-sectional studies are particularly informative for characterizing the clinical phenotype of AD in relation to personality, whereas longitudinal studies better capture personality as a risk-modifying factor across the life course (see Figure 2).

**Table 3:** Characteristics of the included cross-sectional studies and associations between personality traits and mild cognitive impairment (MCI)/Alzheimer’s disease (AD) risk.

Study	Sample	Tools	NEU	EXT	OPE	AGR	CON
Pocnet et al. (239)	*n* = 54 (AD), *n* = 64 (controls)	NEO-PI-R	↑	↓	↓	—	↓
Donati et al. (44)	*n* = 90 (CN) *n* = 63 (MCI)	NEO-PI-R	↑	↓	↓	—	—
Duchek et al. (59)	*n* = 436 CN, *n* = 74 dementia	NEO-FFI	↑	—	—	—	↓
Rouch et al. (117)	Prodromal AD: *n* = 118 mild AD: *n* = 63	NEO-FFI	—	—	—	—	—
Roy et al. (240)	Healthy *n* = 63 a-MCI probable AD possible AD *n* = 119	NEO-FFI	—	—	↓	—	—

↑, higher risk/unfavorable outcome; ↓, lower risk/protective effect; —, no association/no data N/A, not applicable. NEO-PI/NEO-PI-R, NEO Personality Inventory (Revised); NEO-FFI, NEO Five-Factor Inventory; MIDI, Midlife Development Inventory; EPI, Eysenck Personality Inventory; Mini-Markers, Saucier’s Mini-Markers. NEO trait abbreviations: NEU, Neuroticism; EXT, Extraversion; OPE, Openness to experience; AGR, Agreeableness; CON, Conscientiousness.

**Table 4:** Characteristics of the included longitudinal studies and associations between personality traits and mild cognitive impairment (MCI)/Alzheimer’s disease (AD)/dementia/risk.

Study	Sample	Tools	NEU	EXT	OPE	AGR	CON
AD/dementia							
Duberstein et al. (100)	*n* = 767 (>6 years)	NEO-FFI	↑	—	↓	—	↓
Terracciano et al. (39)	*n* = 1,671 (up to 22 years)	NEO-PI-R	↑	—	—	—	↓
Terracciano et al. (46)	*n* = 2046 (up to 36 years)	NEO-PI	↑	—	—	↓	↓
Aschwanden et al. (38)	*n* = 6887 (ELSA)/2,778 (HILDA)	MIDI/Mini-Markers	↑	↓	↓	—	↓
Crowe et al. (37)	*n* = 4,039 (25 years)	EPI	↑ (interaction effects with extraversion)	↓ (moderate); ↑ (low/high NEU interaction)	—	—	—
Johansson et al. (41)	*n* = 800 women (38 years)	EPI	↑	↑ N + ↓ E → highest AD dementia risk	—	—	—
Luchetti et al. (241)	*n* = 13,987 (4 years)	MIDI	↑	↓	↓	—	↓
Dar-Nimrod et al. (55)	*n* = 602	NEO-FFI	↑	↑ (interaction APOE4)	—	—	—
Nishita et al. (242)	*n* = 594	NEO-FFI	—	—	↓	—	↓
Strickhouser and Sutin (243)	*n* = 9,899	MIDI	↑	—	↓	—	↓
Terracciano et al. (4)	*n* = 111	NEO-PI-R	↑	—	—	—	↓
Terracciano et al. (5)	*n* = 1,668	NEO-PI-R	↑	—	↓	↓	↓
Wilson et al. (30)	*n* = 939	NEO-FFI	—	—	—	—	↓
MCI							
Ayers et al. (244)	*n* = 524	BFI	↑	—	—	—	—
Kuzma et al. (45)	*n* = 221	NEO-FFI	↑	—	—	—	—
Wilson et al. (30)	*n* = 939	NEO-FFI	—	—	—	—	↓
Yoneda et al. (47)	*n* = 785	IPIP	↑	—	—	—	—
Yoneda et al. (48)	*n* = 1954	NEO-FFI	↑ (higher risk: NCI → MCI; ↓ likelihood: MCI → NCI)	↑ (MCI → NCI)	—	—	↓ (lower risk: NCI → MCI)

↑ = increased risk/higher likelihood of cognitive decline or dementia; ↓ = protective effect/reduced risk; — = no significant association reported. Direction of associations is indicated such that higher levels of a given trait reflect its relationship with dementia risk, for example: higher neuroticism (N) is associated with increased risk, whereas higher conscientiousness (C), extraversion (E), and openness (O) are generally associated with reduced risk. NEO-PI/NEO-PI-R = NEO Personality Inventory (Revised); NEO-FFI = NEO Five-Factor Inventory; MIDI = Midlife Development Inventory; BFI = Big Five Inventory; BFFQ = Big Five Factor Questionnaire; IPIP = International Personality Item Pool; EPI = Eysenck Personality Inventory; MCI = Mild Cognitive Impairment; NCI = No Cognitive Impairment. NEO trait abbreviations: NEU, Neuroticism; EXT, Extraversion; OPE, Openness to experience; AGR, Agreeableness; CON, Conscientiousness.

In addition, Table 5 summarizes the broader integrative framework linking personality dimensions to AD, encompassing cognitive outcomes, brain structural differences, and underlying biological pathways. This synthesis highlights that personality-related risk is not limited to behavioral or cognitive manifestations but extends to neurobiological mechanisms, including regional brain morphology, functional activation patterns, and neurochemical systems implicated in AD pathophysiology.

**Table 5:** Personality dimensions and Alzheimer’s disease risk: cognitive outcomes, brain structure, and biological pathways.

Personality trait	Cognitive functions	AD/MCI risk	Neurobiological & biomarker correlates (AD context)
NEU↑ (risk)	↓ Memory; ↓ Executive functions; ↑ Cognitive decline; ↑ subjective complaints (72–74)	↑ AD risk; ↑ MCI risk; ↑ dementia incidence; possible prodromal marker (38, 39, 43, 45, 47)	↓ Hippocampal & prefrontal volume; ↑ tau (entorhinal cortex, amygdala); ↑ WMH; Faster atrophy (57–59, 64) ↑ inflammation; ↓ CSF Aβ42; ↑ tau; (2, 54, 71)
EXT ↕ (context-dependent)	↑ cognitive abilities (older adults); mixed/context-dependent effects (84)	↓ AD risk (small effect);protective at moderate levels; interaction with neuroticism(37, 39, 42)	↓ stress & depression risk; cortisol regulation (8, 86, 88, 90)
CON↑ (protective)	↑ cognitive performance; ↓ cognitive decline; resilience to impairment (46, 91)	↓ AD risk; ↓ MCI risk; ↓ conversion risk; independent of APOE ε4 (38, 39, 59)	↑ brain volume (PFC, hippocampus); ↑ white matter integrity; ↓ WMH; slower atrophy (57, 93, 238) ↓ CSF tau; ↓ inflammation (CRP, IL-6); healthier lifestyle mediation; (3, 54, 94)
OPE ↑ (protective)	↑ memory; ↑ executive functions; ↑ processing speed; ↑ cognitive reserve (102–105)	↓ AD risk; ↓ dementia risk; ↓ MCI risk (38, 39, 44, 45, 100)	↑ cognitive reserve; ↑ neural plasticity via engagement; mixed direct biomarker evidence (11, 71)
AGR (indirect)	Indirect via social functioning & emotional regulation (109)	Mixed/weak evidence; small or non-significant effect (39, 106, 107)	Indirect behavioral pathways; no consistent biomarker associations

AD, Alzheimer’s disease; MCI, mild cognitive impairment; PFC, prefrontal cortex; WM, white matter; WMH, white matter hyperintensities; WML, white matter lesions; CSF, cerebrospinal fluid; Aβ42 (Aβ1–42), amyloid beta 42; Tau, tau protein; p-tau, phosphorylated tau; CRP, C-reactive protein; IL-6, interleukin-6; APOE ε4, apolipoprotein E ε4 allele. NEO trait abbreviations: NEU, Neuroticism; EXT, Extraversion; OPE, Openness to experience; AGR, Agreeableness; CON, Conscientiousness.

### Personality dimensions and modifiable risk factors for dementia

In the present review, we also aimed to consider the role of personality traits in the context of modifiable risk factors for dementia (67). Increasing evidence suggests that personality may influence dementia risk not only directly but also indirectly through lifestyle, health behaviors, and the course of somatic diseases.

Personality traits are associated with physical functioning, with neuroticism acting as a risk factor, whereas extraversion, openness, and conscientiousness appear protective (112). A large longitudinal study demonstrated that both baseline levels and changes in personality traits—particularly conscientiousness and neuroticism—predict multiple physical health outcomes (113). At the same time, the onset of chronic diseases is linked to declines in extraversion, emotional stability, conscientiousness, and openness, with stronger effects observed as disease burden increases (114). Personality also plays a key role in health-related behaviors. Data from over 28,000 participants indicate that extraversion, conscientiousness, and openness promote the initiation and maintenance of physical activity, whereas neuroticism increases the likelihood of cessation; notably, agreeableness was also associated with more favorable outcomes (115). Similar patterns are observed in smoking behavior—smokers tend to have higher extraversion and neuroticism and lower conscientiousness; smoking initiation is associated with extraversion and low conscientiousness, while relapse is linked to neuroticism (116).

Personality is also relevant for treatment-related behaviors. Higher neuroticism is associated with an increased risk of medication non-adherence (117) and a higher likelihood of polypharmacy, whereas conscientiousness and extraversion show protective effects (118). Furthermore, low conscientiousness and, to a lesser extent, low emotional stability are associated with increased mortality risk from coronary heart disease and stroke, independent of baseline disease and health behaviors (119).

Evidence regarding intervention outcomes remains less consistent. A systematic review suggests that the role of personality in intervention efficacy is inconclusive, although openness—and to some extent conscientiousness—may be associated with better outcomes (120).

From a clinical perspective, these findings support the use of personality traits in early risk identification and dementia care planning (71). At the same time, personality is gaining increasing attention as a factor in successful aging, although current findings remain fragmented and require further integration (121). Beyond clinical applications, these findings also have broader implications at the public health, educational, and policy levels, suggesting that integrating personality-informed approaches into health promotion and prevention programs may support more effective reduction of dementia risk (11, 67). Importantly, accumulating evidence indicates that personality traits are modifiable through targeted interventions, with effects that can persist over time, although the extent of change may depend on the specific trait and individual engagement in the change process (122–124). For example, a cognitive training program increased openness to experience in older adults, highlighting the potential for intervention-based change (125).

In the previous sections of this article, we referred to the concept of cognitive reserve as a key factor explaining why individuals differ in their cognitive resilience to underlying brain pathology. Recent findings suggest that personality may represent an important, yet still underexplored, contributor to this mechanism. Higher openness has been associated with greater cognitive reserve, potentially buffering the impact of neuropathology on cognitive functioning, whereas neuroticism does not appear to show such associations in healthy populations (126). This perspective is further supported by a recent meta-analysis of longitudinal studies, which showed that cognitive reserve accumulated across the lifespan significantly reduces the risk of dementia, with the strongest protective effects observed in early and late life. Importantly, the findings also highlight the role of social connection as a potential pathway for enhancing cognitive reserve and lowering dementia risk (127).

### Personality metatraits as a bridge between trait psychology and neuroscience

Recent years have seen a growing number of systematic reviews and meta-analyses on the association between Big Five traits and cognitive decline, reflecting increasing recognition of personality’s role in shaping cognition through both direct (e.g., neural mechanisms) and indirect (e.g., lifestyle) pathways (40, 107). Personality neuroscience posits that stable individual differences in behavior, emotion, and cognition must proximally reflect variation in brain function, irrespective of their distal genetic or environmental determinants. Personality traits are therefore conceptualized as probabilistic patterns in the frequency and intensity of affective, motivational, and cognitive states (128–131). Converging evidence from behavioral genetics supports this position: twin studies demonstrate substantial heritability not only of broad Big Five domains but also of their lower-order facets (approximately 25–65% of reliable variance), with highly similar genetic factor structures observed across North American, European, and East Asian samples (132–134). A meta-analysis of 62 effect sizes from over 100,000 participants showed that roughly 40% of individual differences in personality are genetic and 60% environmental, with study design (twin vs. adoption) affecting heritability estimates (135). Such findings suggest that the five-factor model reflects stable, biologically grounded mechanisms of individual differences.

Previous systematic reviews suggest that considering the joint influence of all five traits within the Big Five framework may offer a more comprehensive understanding of how personality contributes to cognitive aging trajectories (40). Within the hierarchical Big Five framework, accumulated evidence indicates that the five traits are not entirely orthogonal but cluster into two higher order metatraits: Stability (reversed Neuroticism, Agreeableness, and Conscientiousness) and Plasticity (Extraversion and Openness/Intellect) (136–138) (see Table 1; Figure 1). This higher-order structure has been observed across developmental stages and cultures. However, twin and multimethod analyses indicate that these Stability/Plasticity factors likely reflect a combination of substantive shared variance and evaluative bias rather than fully distinct biological entities (139). Accordingly, metatraits should be interpreted as higher-order statistical regularities that may correspond to partially overlapping neurobiological systems.

Stability has been hypothesized to relate primarily to serotonergic function, reflecting the capacity to maintain goal representations, regulate affect, and inhibit disruptive impulses. Molecular evidence linking serotonergic polymorphisms (e.g., 5-HTTLPR) to affective reactivity and neuroticism, along with neuroimaging findings implicating fronto-limbic circuits in emotional regulation, provides indirect support for this account (86, 140). In a large cohort of older adults (*n* > 550), higher Stability was significantly associated with greater white matter integrity (fractional anisotropy) in the left uncinate fasciculus, fully accounting for the effect observed for Conscientiousness. These findings suggest that Stability reflects microstructural variation in fronto-limbic white matter pathways (141). At the neurochemical level, serotonin modulates excitation–inhibition balance in the prefrontal cortex (PFC) through receptor-specific effects on glutamatergic and glutamatergic and *γ*-aminobutyric acid (GABA)-ergic transmission, thereby influencing cognitive control and emotion regulation (142).

Plasticity, in turn, has been linked predominantly to dopaminergic function and reflects the tendency to generate new goals, interpretations, and behavioral strategies—that is, to engage in exploration. This interpretation is supported by pharmacological evidence connecting agentic extraversion to dopaminergic D2 receptor sensitivity, as well as neuroimaging findings linking Extraversion to reward-related regions such as the medial orbitofrontal cortex and ventral striatum, and Openness/Intellect to the global efficiency of the default mode network (29, 86, 143–145). In the PFC, dopamine regulates goal-directed processing, optimizing working memory and cognitive control, while interacting dynamically with norepinephrine, which tunes cortical function in relation to arousal and stress (142). At the circuit level, dopaminergic signaling modulates pyramidal neurons and GABAergic interneurons in the PFC, shaping excitatory–inhibitory microcircuits (142). In the hippocampus, dopamine acting on D1/D5 receptors enhances synaptic plasticity and memory encoding. Notably, novelty-induced enhancement of episodic-like memory depends critically on tyrosine hydroxylase–positive neurons of the locus coeruleus (LC), which co-release dopamine in the hippocampus (146, 147). Importantly, Plasticity refers to adaptive psychological flexibility and should not be conflated with synaptic or structural neural plasticity (144).

**Figure 3:**
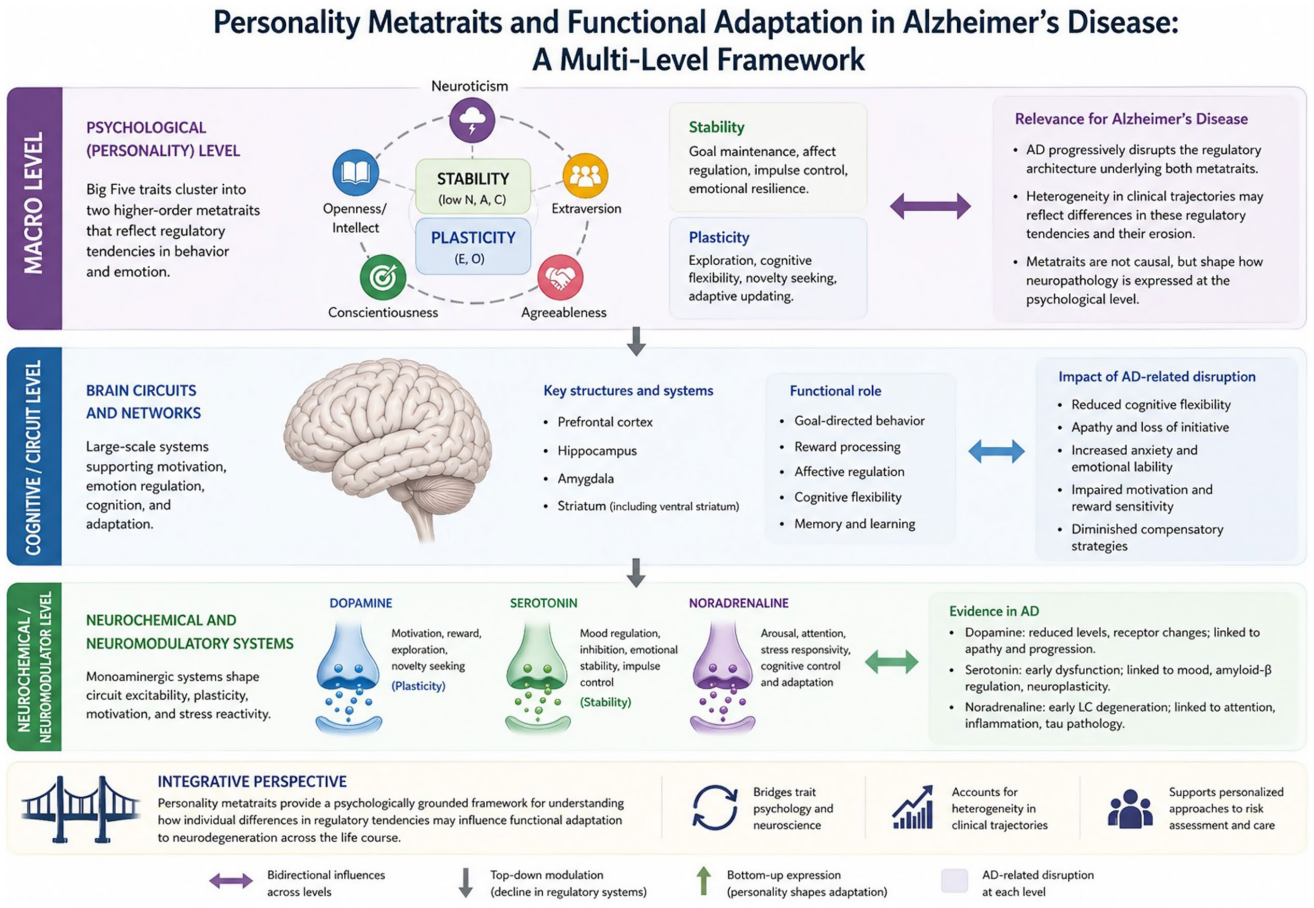
Personality metatraits and Alzheimer’s disease: a multi-level neurobehavioral framework.

These interpretations are formalized within the Cybernetic Big Five Theory, which conceptualizes personality traits as emergent properties of evolved self-regulatory systems. From a cybernetic perspective, the brain operates as a goal-directed control system that monitors discrepancies between current and desired states and adjusts behavior through feedback mechanisms (29). Within this framework, serotonergic and dopaminergic systems can be understood not as isolated trait “causes,” but as components of broader regulatory architectures governing stability of goal pursuit and exploration under uncertainty. Dopamine has been consistently implicated in reward valuation, incentive salience, and exploratory motivation, whereas noradrenergic and serotonergic systems contribute to threat sensitivity and behavioral inhibition (29, 128, 148). Taken together, this neurobiological perspective provides a conceptual bridge between hierarchical models of personality and large-scale neuromodulatory systems that undergo age-related alterations and degeneration in AD. Rather than reducing personality traits to single neurotransmitters, the metatrait framework offers a systems-level account of how variation in regulatory architecture may shape adaptive functioning—and its disruption—in the context of neurodegeneration. Reference to serotonin and dopamine was not intended to be reductionistic; rather, the aim was to build a framework that connects different levels of explanation and complexity. Contemporary neuroscientific explanations are inherently multi-level: descriptions at a single level allow for horizontal explanations, whereas transitions across levels within a theoretical hierarchy provide vertical explanations (see Figure 3). Such a perspective may offer a more integrative account of brain–behavior relationships (149).

## Monoaminergic systems in Alzheimer’s disease: a translational perspective from personality metatraits

### Dopamine

Converging evidence indicates that dopaminergic dysfunction is implicated in AD. A meta-analysis confirmed reduced dopamine levels and receptor alterations in AD (150). Grey matter loss in AD was negatively associated with serotonergic and presynaptic dopaminergic markers, particularly in temporo-frontal regions. The strength of these associations was modulated by AD polygenic risk scores, independent of APOE effects (151), and prodromal AD is characterized by reduced [^123^I]FP-CIT (^123^I-ioflupane) binding—a radioligand used in SPECT (Single-Photon Emission Computed Tomography) imaging to assess dopamine transporter availability—and disrupted mesocorticolimbic connectivity, while the nigrostriatal pathway remains relatively preserved (152). A meta-analysis of PET and SPECT studies in healthy adults (95 studies including 2,611 participants) demonstrated moderate-to-large age-related reductions in dopamine receptors and transporters, while synthesis capacity remained relatively preserved (153), and neuropathological data indicate degeneration of ventral tegmental area neurons contributing to memory and reward dysfunction (154, 155). Dopaminergic alterations are also linked to apathy and other neurobehavioral symptoms (156), and sex-specific reductions in D2 receptors may partly account for differential vulnerability in women (150).

The link between extraversion and cognition may be explained by dopaminergic activity, which is reduced in prefrontal regions even in early AD and relates to executive deficits and apathy. From a translational perspective, apathy may represent a clinical manifestation of reduced Plasticity-related dopaminergic signaling. Robust longitudinal evidence indicates that apathy strongly predicts progression from MCI to AD (157–159), independently of depression (157, 158). It is considered an early marker of accelerated functional decline and increased mortality (160). Thus, apathy may reflect disruption of motivational-exploratory systems that, at the psychological level, align with diminished Plasticity. In contrast, higher baseline dopamine levels in healthy individuals are associated with greater extraversion, suggesting a more reactive reward system (40). Another review highlights a complex relationship between dopamine function (particularly D2 receptors) and social behavior, with effect directions depending on measurement methods and potentially influenced by endogenous dopamine levels (161).

### Serotonin

Serotonin (5-HT) plays a central role in cognitive functioning through its interactions with cholinergic, glutamatergic, GABAergic, and dopaminergic systems, as well as its regulation of prefrontal excitatory–inhibitory balance and synaptic plasticity (142, 162). Converging clinical, postmortem, and neuroimaging evidence indicates that serotonergic dysfunction emerges early in AD and is associated with both cognitive and affective symptoms (163). PET studies in cognitively normal individuals suggest that enhanced 5-HT signaling is linked to lower amyloid-*β* accumulation, a finding supported by retrospective analyses of antidepressant-treated patients (163). Serotonin dysregulation contributes to amyloid-β and tau pathology, mitochondrial dysfunction, impaired mitophagy, vascular alterations, and reduced neuroplasticity (164, 165). Accordingly, modulation of serotonergic transmission—particularly via specific 5-HT receptor subtypes—is increasingly considered a potential disease-modifying strategy rather than merely symptomatic treatment (166, 167). Notably, much of the current evidence derives from integrative reviews rather than large-scale primary studies with clearly quantified effect sizes, underscoring the need for further empirical validation.

From the perspective of personality metatraits, serotonergic dysfunction may be interpreted—at a translational level—as compromising mechanisms related to Stability, namely sustained goal maintenance and resistance to emotional disruption. Importantly, this interpretation extends beyond the original psychological operationalization of Stability and refers here to neurobiological regulatory processes rather than trait scores per se.

### Noradrenaline

The locus coeruleus–noradrenaline (LC–NA) system is among the earliest sites of neurodegeneration in AD and represents a critical yet underexplored contributor to disease progression (168–170). Early degeneration of LC neurons leads to NA depletion, which promotes neuroinflammation through impaired adrenergic regulation of glial cells and contributes to synaptic dysfunction and cognitive decline (168, 171, 172). Noradrenergic dysfunction is detectable already at prodromal stages, where amyloid-β alters α2-adrenergic receptor signaling and contributes to loss of prefrontal synaptic proteins critical for cognitive control (142). Noradrenaline exerts complex and bidirectional effects on AD pathology. Adrenergic receptor activity modulates both production and clearance of amyloid-β and tau, and NA depletion is associated with increased neuroinflammation, enhanced amyloid and tau burden, impaired synaptic plasticity, and mitochondrial dysfunction (172, 173). Experimental models further demonstrate that selective NA loss induces working memory deficits and increases pathological phosphorylation of tau and TDP-43 (TAR DNA-binding protein 43) in cortical and hippocampal regions, supporting a direct link between LC degeneration and proteinopathy (174). At the network level, LC functional connectivity correlates with amyloid and tau burden in amyloid-positive individuals; importantly, higher catecholamine synthesis capacity appears to buffer pathology-related hyperactivity (175). Neurochemical studies additionally indicate increased NA turnover and dopaminergic imbalance in frontal regions, suggesting broader catecholaminergic dysregulation related to LC and ventral tegmental area degeneration (176).

Clinically, early noradrenergic dysfunction affects attention, working memory, and neuropsychiatric symptoms (177). Given NA’s central role in stress responsivity, chronic stress and elevated glucocorticoids may further exacerbate LC vulnerability and accelerate disease onset, with evidence for sex-related differences in risk (178). Finally, noradrenergic activity may constitute a biological substrate of cognitive reserve. Lifelong intellectual engagement and environmental enrichment, through repeated activation of the LC–NA system, may enhance neuroprotective and compensatory mechanisms, potentially delaying the clinical manifestation of AD pathology (179).

### Integrating personality metatraits with functional adaptation in Alzheimer’s disease

Within the metatrait framework, Stability and Plasticity describe two broad dimensions of behavioral and emotional organization, reflecting regulatory tendencies in goal maintenance and affective control (Stability) versus exploration, cognitive flexibility, and adaptive updating in response to novelty (Plasticity) (129, 137, 138). Importantly, these constructs refer to probabilistic styles of functioning (128, 130) rather than structural neural plasticity or direct biomarkers of disease. From this perspective, AD can be conceptualized as progressively disrupting the regulatory architecture underlying both metatraits. Early neurodegenerative changes may simultaneously affect episodic memory, cognitive flexibility, and motivational processes (12, 180, 181), contributing to a weakening of Plasticity-related functioning—thereby limiting the individual’s capacity to generate alternative strategies, engage with uncertainty, or compensate for emerging deficits. Clinically, this may manifest as apathy and reduced initiative—symptoms associated with dopaminergic dysfunction and accelerated progression from MCI to AD (157–159). At the same time, affective dysregulation and heightened anxiety (182, 183) may reflect erosion of mechanisms aligned with Stability, particularly those supporting sustained goal-directed behavior under stress (144). Elevated anxiety—a facet of neuroticism—has been identified as a strong predictor of progression to MCI in Aβ-positive individuals, reinforcing its potential as an early clinical marker (184). Consistent with this interpretation, a personality profile characterized by relatively high activity levels and emotional calmness has been associated with a lower risk of dementia and interpreted as reflecting a more mature or adaptive personality configuration (185). In a longitudinal clinical–neuropsychological–genetic study (*n* = 212, ≥2-year follow-up), poorer verbal memory predicted progression from subjective cognitive decline/MCI to AD, while lower emotional stability and lower intellectual engagement were associated with a higher risk of conversion to MCI (186).

Crucially, this interpretation does not imply that metatraits cause AD. Rather, they may shape the way neuropathology is expressed at the psychological level, consistent with pathoplastic and reserve-based models of dementia (8). Individuals higher in Plasticity may initially demonstrate more flexible coping and compensatory strategy use in response to subtle decline, whereas higher Stability may buffer against stress-related symptom amplification. Conversely, progressive degeneration of large-scale neuromodulatory systems implicated in motivational and regulatory processes may gradually undermine these adaptive capacities, contributing to heterogeneity in clinical trajectories (142, 170). Thus, metatraits provide a psychologically grounded framework for interpreting variability in functional adaptation to neurodegeneration. By integrating hierarchical personality models with systems-level neuroscience of aging and dementia (129, 180), this approach bridges trait psychology and translational neurobiology without reducing personality constructs to direct neurobiological proxies (see Figure 3). However, despite our efforts, we were unable to identify studies on AD that explicitly incorporate personality metatraits; this gap may represent an opportunity to revisit existing data from a novel, more integrative perspective.

### Metatraits or facets? Integrating levels of personality description in dementia research

Although the present review emphasizes higher-order metatraits as a useful framework for linking personality structure with neurobiological systems, it is important to acknowledge that earlier empirical research has consistently demonstrated the relevance of lower-order personality facets. Facets represent more specific and behaviorally proximal components of personality domains and may therefore capture individual differences with greater precision than broad traits or metatraits (9, 187). From this perspective, metatraits can be understood as organizing principles, whereas facets provide finer-grained resolution of psychological functioning.

Recent large-scale evidence supports the importance of this multilevel approach. A population-based neuroimaging study from the UK Biobank (*n* = 298,259) showed that specific trait-like characteristics—such as nervousness, warmth, diligence, sociability, and curiosity—were differentially associated with brain health, implicating frontal and temporal regions as well as biological pathways related to inflammation and lipid metabolism (188). Notably, these constructs map more closely onto facet-level expressions than broad domains, suggesting that biologically meaningful variation may be better captured at this level of analysis.

Similarly, longitudinal and meta-analytic findings indicate that personality changes in neurodegenerative conditions occur not only at the domain level but also within specific facets. An informant-based study combined with a meta-analysis (18 samples, *n* = 542) demonstrated substantial premorbid-to-current shifts in individuals with MCI and dementia, particularly declines in conscientiousness (e.g., self-discipline, competence) and extraversion (e.g., energy, assertiveness), alongside increases in neuroticism (e.g., vulnerability to stress) (189). These findings highlight that clinically relevant personality changes are often driven by alterations in specific functional components rather than global trait shifts. In the Lifelines study (*n* = 79,911), associations between personality and cognitive functioning were most pronounced at the facet level. Higher hostility and vulnerability (neuroticism), as well as lower excitement seeking (extraversion) and competence (conscientiousness), were associated with poorer cognitive performance, whereas other facets showed weaker or differential effects. These associations were generally small, largely independent of lifestyle factors, and varied across age groups, underscoring the context-dependent nature of personality–cognition links (187). Informant-rated changes in AD were observed across specific personality facets, including increased Anxiety (N1), and changes in Assertiveness (E3) and Activity (E4), as well as Depression, Vulnerability, and Openness/Ideas, with premorbid personality emerging as the strongest predictor of most reported changes, while education, disease duration, and gender influenced selected facets (190).

Furthermore, facet-level analyses of neuroticism consistently highlight anxiety, stress vulnerability, and depression as key components associated with increased risk of AD and faster cognitive decline, particularly in episodic and working memory, independently of classical neuropathological markers (93). These findings are supported by neuropathological evidence showing that such facets are linked to more advanced neurofibrillary pathology and a reduced likelihood of remaining asymptomatic despite AD pathology (4). Neuroimaging studies further indicate that the depression facet is associated with alterations in amygdala microstructure, potentially affecting emotional information processing (60).

For conscientiousness, facet-level findings are particularly robust. Facets such as dutifulness (responsibility), self-control, and industriousness are consistently associated with lower risk of cognitive impairment and dementia (9). In contrast, lower levels of order and competence have been linked to a higher likelihood of clinical dementia despite the presence of AD neuropathology, suggesting that conscientiousness contributes to resilience rather than directly reducing pathological burden (4). This interpretation is further supported by evidence that conscientiousness modifies the relationship between neuropathology and cognitive outcomes, consistent with a cognitive reserve framework (30). In the case of extraversion, sociability has been linked to greater hippocampal volume, while activity and positive affect show less consistent associations; other studies, however, suggest that activity and positive emotions may relate to lower cognitive impairment scores (191). Evidence for agreeableness remains more limited but also points to facet-specific effects, with traits such as trust and modesty associated with reduced risk of cognitive impairment (5, 192). At the same time, some previously implicated facets show inconsistent or null findings, highlighting variability across studies (9, 39).

Taken together, these findings suggest that metatraits and facets should not be viewed as competing frameworks but rather as complementary levels of analysis. Metatraits offer a parsimonious, systems-level perspective aligned with large-scale neurobiological models, whereas facets provide the specificity necessary to capture heterogeneous behavioral and clinical manifestations of cognitive aging and dementia. Integrating both levels may therefore offer the most comprehensive account of how personality shapes vulnerability and resilience in neurodegenerative processes (see Figure 3).

### Beyond the big five: alternative neurobehavioral frameworks

The Big Five framework derives from the lexical hypothesis, according to which fundamental personality traits are encoded in natural language and can be identified through statistical analyses of trait-descriptive adjectives (193, 194). While this descriptive approach has demonstrated strong predictive validity in dementia research, it does not specify underlying neurobiological mechanisms. Complementary models attempt to address this limitation.

One influential, though partly hypothetical, framework is Gray’s Reinforcement Sensitivity Theory (RST) (148, 195, 196). Gray (195) proposed a reconceptualization of Eysenck’s personality dimensions by integrating evidence from neuropharmacology and brain lesion studies in animal models. Central to his framework was the idea that approach and avoidance behaviors are governed by distinct neurobiological systems. This led to the formulation of the RST, which posited two core systems: The Behavioral Activation System (BAS), associated primarily with striatal and thalamic regions and mediating approach behavior and reward sensitivity, and the Behavioral Inhibition System (BIS), involving septo-hippocampal circuits and underlying responses to conflict and punishment signals. Based on the balance between BIS and BAS activity, Gray (148) proposed two primary personality dimensions: anxiety and impulsivity. Anxiety reflects heightened sensitivity of the BIS, leading to increased avoidance and vigilance in response to potential punishment or novelty. Impulsivity, in contrast, reflects dominance of the BAS, associated with approach behavior and sensitivity to reward. RST thus offers a mechanistic, bottom-up alternative to descriptive, language-based taxonomies such as the Big Five, by anchoring personality variation in neural systems mediating approach–avoidance and conflict processing. In their revised model (r-RST), Gray and McNaughton (196) further refined this theory, distinguishing between the neural substrates of anxiety and fear, which correspond to activity in the BIS and the Fight–Flight–Freeze System (FFFS), respectively. The RST framework captures variance at a more fundamental neurobehavioral level—potentially explaining why facets such as anxiety, vigilance, and reward sensitivity show clearer neurobiological and cognitive correlates than aggregate trait scores (196, 197). The BAS has been consistently linked to higher impulsivity and positive affect, as well as a range of approach-related behaviors, including substance use, aggression, and binge–purge tendencies. In contrast, the BIS shows strong associations with neuroticism, negative affect, and internalizing psychopathology, particularly anxiety and depression (198–204). Dopaminergic pathways (e.g., DRD2, catechol-O-methyltransferase-COMT) have been shown to influence BIS/BAS sensitivity, stress reactivity, and emotional regulation (205, 206). Neuroanatomical studies provide partial support for RST-consistent substrates, linking fear-related processes to anterior cingulate cortex volume, BAS to caudate volume, and BIS to posterior cingulate cortex and hippocampal lateralization (207). A functional magnetic resonance imaging (fMRI) study showed that trait anxiety within RST is associated with resting-state activity in the amygdala and hippocampus, supporting their role in the BIS (208). Individuals with heightened BIS sensitivity—associated with anxiety, vigilance, and punishment sensitivity—may experience prolonged HPA axis activation and increased allostatic load, mechanisms linked to hippocampal atrophy and accelerated cognitive decline (70). The hippocampus, rich in GABAergic interneurons essential for memory and inhibitory control, is particularly sensitive to chronic stress, which impairs GABAergic modulation and glucocorticoid feedback, plausibly linking emotional reactivity to episodic memory dysfunction (209, 210). A systematic review of 22 studies including nearly 500,000 participants found that higher stress is associated with increased dementia risk, suggesting that personality traits linked to stress vulnerability may indirectly contribute to disease risk (211). Research on mild behavioral impairment (MBI) links emergent affective symptoms (anxiety, irritability) — which align conceptually with elevated BIS/neuroticism — to higher dementia risk (212). Conversely, higher BAS sensitivity—associated with reward responsiveness, goal pursuit, and sustained motivation—may promote engagement in cognitively stimulating and health-protective activities, paralleling the protective effects of conscientiousness observed in epidemiological research (25, 213).

While Gray’s RST appears useful for interpreting the emotional–motivational substrates of neuroticism, its applicability to conscientiousness—a trait consistently linked with lower AD risk—remains less straightforward. Evidence indicates that conscientiousness correlates positively with aspects of executive functions related to mental set-shifting and multicomponent behavioral organization, rather than with prepotent response inhibition or simple response suppression (213, 214). Neuroimaging studies further link higher conscientiousness with greater grey matter volume in the frontoparietal network and inferior frontal gyrus (IFG)—regions implicated in top-down cognitive control and goal maintenance (215, 216). These findings suggest that conscientiousness reflects a capacity for sustained, deliberate control of behavior rather than rapid inhibition of automatic responses, aligning it more closely with prefrontal executive regulation than with the BIS/BAS framework of RST. In this view, conscientiousness may serve as a cortically mediated self-regulatory system, supporting stable behavioral control and adherence to long-term goals, rather than as a direct counterpart to the anxiety-driven BIS. Consequently, while neuroticism may index emotional vulnerability through limbic–HPA pathways, conscientiousness likely confers resilience through frontoparietal executive networks that underlie planning, organization, and self-regulation—mechanisms that could contribute to preserved cognitive function in aging and protection against AD. Although direct applications of RST to AD remain limited, the model provides a useful interpretative lens for existing personality–AD findings. Some studies report higher BIS scores or altered BIS/BAS profiles in AD or frontotemporal dementia (217).

Psychometric tools derived from this theory—such as the BIS/BAS scales (218) and their subsequent revisions, including the r-RST questionnaire assessing BIS, BAS, and FFFS—offer valuable means to examine how motivational and neurobiological processes link personality dimensions with vulnerability to MCI and AD (219). These instruments allow for the assessment of individual differences in behavioral inhibition and activation, providing an operational bridge between motivational systems and neurobiological vulnerability. Although the revised RST has prompted the development of several updated psychometric tools (57, 206, 220, 221), consensus regarding the optimal structure—particularly of the BAS and FFFS scales—remains unresolved. Recent work has sought to refine these measures and align them with the tripartite RST framework, demonstrating promising factorial stability and theoretical validity (222–224).

Proposing Gray’s model does not entail rejecting the Big Five or questioning its relevance. Rather, it highlights the contextual and methodological limits of trait models developed primarily to describe normative personality variation in healthy populations, rather than to address clinical neurodegenerative conditions. Historically, personality psychology focused on describing how individuals differ rather than explaining why they differ, partly due to limited neuroscience at the time (225). Advances in neuroimaging and molecular genetics now make it possible to investigate the neural substrates underlying stable individual differences. While RST has not yet been as widely applied to AD as the Big Five personality framework, the available studies show promising signals. Thus, the model is *testable and under investigation.*

Other frameworks, such as the Affective Neuroscience model (226, 227) and computational personality models (228–231), offer additional perspectives on motivational and affective systems. However, these models have been only minimally applied in Alzheimer’s research to date.

### Limitations

In interpreting the findings of this review, several limitations must be acknowledged. First, this work is not a meta-analysis; rather, it provides a narrative synthesis of the literature, and thus we cannot derive quantitative estimates of effect sizes or formally test for publication bias. Second, care must be taken when interpreting personality differences as true *changes* in personality over time. Most of the studies discussed are cross-sectional comparisons between groups, or they test whether baseline differences predict future (e.g., cognitive) change (see Tables 3, 4). Third, the overlap between personality traits (notably neuroticism) and distress or depressive symptoms complicates interpretation of findings—i.e., higher neuroticism scores may partly reflect transient distress rather than enduring personality dispositions (78). Moreover, many studies focus on genetic correlations of personality traits but fail to link these traits robustly with biomarker or neuroimaging endpoints. One explanation for this discrepancy is that while personality traits may reflect broad, pleiotropic genetic influences contributing to vulnerability (e.g., a *general liability* factor), the downstream biological pathways [e.g., brain structure, amyloid/tau burden (13, 16)] may be more distal, non-specific, or influenced by environmental and epigenetic modifiers. In other words, strong genetic associations can exist without clear biomarker correlates because the intermediate mechanisms are heterogeneous and multifactorial (52, 232). Furthermore, personality development reflects complex interactions between genetic and environmental factors that jointly shape individual trajectories. Individuals partly select environments based on heritable traits, and these environments can, in turn, reinforce or modify personality over time (233). Trait stability increases until midlife and declines in older age, with longer assessment intervals associated with lower continuity, highlighting that personality remains a dynamic, lifelong process influenced by both biological maturation and environmental experiences (234). Finally, methodological issues related to personality assessment should also be considered. Some systematic reviews highlight that informant-based personality ratings may introduce bias, suggesting that self-reports from non-demented individuals, including those with MCI, may provide more reliable assessments. This approach allows for a more accurate capture of premorbid personality and reduces potential bias (40).

Furthermore, beyond conventional personality traits, there is also an observed connection between dementia and personality disorders. Notably, personality disorders like obsessive-compulsive personality disorder have been identified as potential contributors to the risk of AD (235). Additionally, other studies suggest a broader association, indicating that nearly all types of personality disorders are linked to an increased risk of dementia (236). Future research should focus on identifying differential patterns of personality change across different types of dementia. It is also necessary to conduct prospective, repeated personality assessments using both self-report questionnaires and assessments by patients’ relatives to better understand the time course of personality changes in the prodromal and clinical phases of dementia (189).

## Conclusion

AD reflects a dynamic interplay between biological vulnerability and enduring personality-related patterns of emotional, motivational, and cognitive functioning. Among the Big Five traits, higher neuroticism and lower conscientiousness have been consistently associated with increased risk of cognitive decline and dementia, partly through stress-related mechanisms, health behaviors, and long-term regulatory processes. Higher neuroticism is linked to stress and unhealthy behaviors (e.g., smoking, inactivity), which indirectly increase the risk of chronic conditions and cognitive decline. In contrast, traits such as conscientiousness and emotional stability support well-being through healthier lifestyles.

Recent advances in personality neuroscience suggest that these associations may also be understood within a broader hierarchical framework of personality organization. Higher-order metatraits—Stability and Plasticity—capture fundamental dimensions of behavioral regulation related to goal maintenance, emotional control, and adaptive flexibility. Emerging evidence linking these dimensions to large-scale neuromodulatory systems, particularly serotonergic and dopaminergic pathways, provides a potential conceptual bridge between trait psychology and the neurobiology of aging. In the context of AD, degeneration of monoaminergic systems—including dopaminergic, serotonergic, and noradrenergic networks—may progressively disrupt motivational, affective, and cognitive regulatory processes that correspond to these metatraits. Such disruptions may contribute to clinical manifestations such as apathy, emotional dysregulation, and reduced adaptive coping with cognitive decline.

Importantly, personality traits and metatraits should not be interpreted as direct causes of AD. Rather, they may influence how underlying neuropathology is behaviorally expressed and how individuals adapt to emerging cognitive changes. Notably, the role of metatraits in AD has not been directly tested to date; therefore, the framework proposed here should be understood as a theoretically grounded and testable hypothesis. Importantly, it can be examined with relatively low methodological cost, including re-analyses of existing datasets using hierarchical models of personality structure.

At the same time, although the present review emphasizes metatraits, it also incorporates evidence at the facet level, acknowledging that lower-order personality components may capture more specific and behaviorally proximal mechanisms. Integrating metatraits, traits, and facets offers a multilevel perspective that may provide a more comprehensive understanding of how personality shapes vulnerability and resilience in AD.

**Table:** 

Considerations for future research Considering higher-order personality metatraits such as Stability and Plasticity may provide a new perspective on existing longitudinal datasets that include Big Five assessments. Because metatraits can be derived from previously collected personality measures, they allow reanalysis of established cohorts and epidemiological studies, potentially offering additional insight into associations with AD-related biomarkers and genetic factors that have not always been consistently detected at the level of individual traits. Future studies should also examine potential sex differences in the associations between personality metatraits and AD-related outcomes, as growing evidence suggests that both personality expression and AD trajectories may vary between men and women. Investigating whether Stability and Plasticity differentially relate to cognitive decline, biomarker profiles, or resilience mechanisms across sexes may provide a more nuanced understanding of vulnerability and protection in aging. Although some findings indicate that age and gender do not consistently moderate personality–cognition associations, evidence from two large samples (*n* = 422 and *n* = 549) and a mini-meta-analysis suggests that gender may specifically moderate the relationship between conscientiousness and cognitive performance, supporting the need for more nuanced, sex-sensitive approaches in this field (237). Another promising direction involves integrating personality assessment with comprehensive neuropsychological evaluation, combining affective–personality dimensions with detailed measures of cognitive functioning (40, 71). Previous research has often examined these domains separately. A more integrative approach could clarify how personality-related affective regulation, motivational processes, and cognitive functioning interact in the context of AD risk and progression.
